# A Real-Time PCR Array for Hierarchical Identification of *Francisella* Isolates

**DOI:** 10.1371/journal.pone.0008360

**Published:** 2009-12-21

**Authors:** Kerstin Svensson, Malin Granberg, Linda Karlsson, Vera Neubauerova, Mats Forsman, Anders Johansson

**Affiliations:** 1 Division of CBRN Defense and Security, Swedish Defense Research Agency, Umeå, Sweden; 2 Department of Clinical Microbiology, Infectious Diseases and Bacteriology, Umeå University, Umeå, Sweden; 3 Central Military Health Institute, Prague, Czech Republic; St. Petersburg Pasteur Institute, Russian Federation

## Abstract

A robust, rapid and flexible real-time PCR assay for hierarchical genetic typing of clinical and environmental isolates of *Francisella* is presented. Typing markers were found by multiple genome and gene comparisons, from which 23 canonical single nucleotide polymorphisms (canSNPs) and 11 canonical insertion-deletion mutations (canINDELs) were selected to provide phylogenetic guidelines for classification from genus to isolate level. The specificity of the developed assay, which uses 68 wells of a 96-well real-time PCR format with a detection limit of 100 pg DNA, was assessed using 62 *Francisella* isolates of diverse genetic and geographical origins. It was then successfully used for typing 14 *F. tularensis* subsp. *holarctica* isolates obtained from tularemia patients in Sweden in 2008 and five more genetically diverse *Francisella* isolates of global origins. When applied to human ulcer specimens for direct pathogen detection the results were incomplete due to scarcity of DNA, but sufficient markers were identified to detect fine-resolution differences among *F. tularensis* subsp. *holarctica* isolates causing infection in the patients. In contrast to other real-time PCR assays for *Francisella*, which are typically designed for specific detection of a species, subspecies, or strain, this type of assay can be easily tailored to provide appropriate phylogenetic and/or geographical resolution to meet the objectives of the analysis.

## Introduction

**Figure 1:**
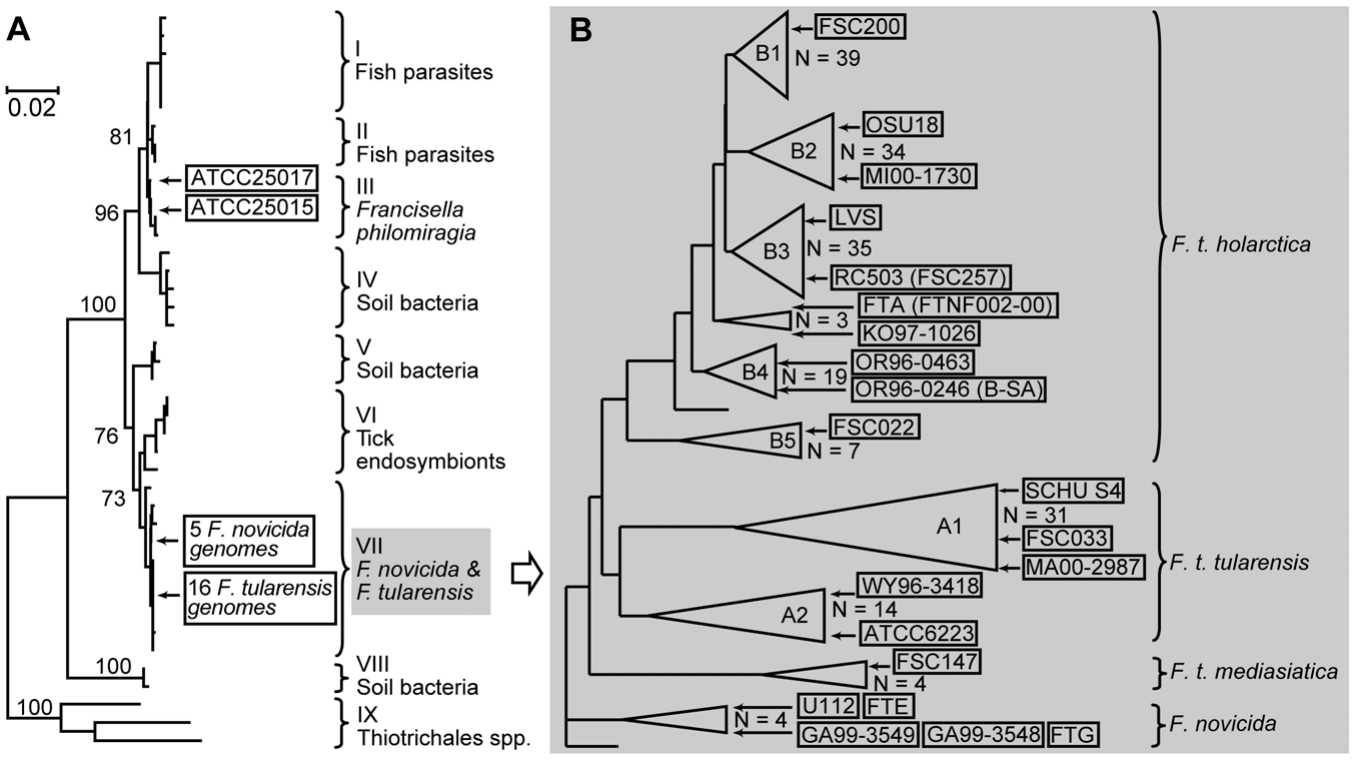
Phylogenies of *Francisella* based on 16S rDNA and MLVA, respectively. A) Phylogeny of *Francisella* and representative relatives based on alignment of 1,070 bp of the 16S rDNA gene. Bootstrap values are indicated at the branching points. The scale bar indicates 0.02 nucleotide changes per site. Modified from [36]. B) Phylogeny of *Francisella* based on MLVA. Subspecies and major genetic branches (A1-A2, B1-B5) are indicated. Currently available genome sequences are in black boxes. Multiple strains are indicated by triangles at the branch edges. Modified from Johansson et al 2004 [26].

The genus *Francisella* consists of three species: *F. philomiragia*, *F. novicida*, and the etiological agent of the zoonosis tularemia, *F. tularensis*. In addition, there are several soil bacteria, tick endosymbionts and fish parasites that are genetically closely related to *Francisella*, but are not (yet at least) assigned to the genus (Figure 1). Three subspecies of *F. tularensis* are recognized, of which *F. tularensis* subspp. *tularensis* and *holarctica* cause severe, sometimes fatal, disease in humans. The third subspecies, *mediasiatica*, is rare and its virulence is described as moderate. *F. tularensis* subsp. *holarctica* has been isolated throughout the northern hemisphere, while *F. tularensis* subspp. *tularensis* and *mediasiatica* are geographically restricted to North America and Central Asia, respectively. The population structure of the two clinically relevant subspecies, *F. tularensis* subsp. *tularensis* (type A) and *F. tularensis* subsp. *holarctica* (type B), is highly clonal, a property that facilitates the design of genetic typing systems and deduction of evolutionary relationships among genetic subclades of *Francisella*, since mutations are mainly inherited vertically [1], [2].

Tularemia is characterized by an acute course of infection, and mortality rates of *F. tularensis* subsp. *tularensis* infections historically reached 5 to 30% before effective antibiotic treatments were available. In contrast, *F. tularensis* subsp. *holarctica* infections are milder and may be fatal only to patients with an impaired immune system [3]. *F. tularensis* can infect humans, via aerosols or the skin, at doses as low as 10 cells [4], [5] and is listed by the CDC as a major potential bioterror agent [6]. Cultivation of *F. tularensis* is often avoided, since it poses considerable risks of laboratory-acquired infections via aerosolization. Laboratory culture work requires biosafety-level 3 (BSL-3) conditions and primary cultivation from a clinical specimen may require a seven-day incubation before colonies visible to the naked eye appear. To shorten the time required for clinical diagnosis, PCR assays targeting 16S rDNA [7] or specific genes encoding outer membrane proteins such as *fopA* [8] and *lpnA* [9]–[11] have been used to detect *Francisella*, and several real-time PCR assays have been developed recently that appear to be more sensitive than conventional PCR [12]–[17]. However, a serious drawback of PCR-detection is that cross-reactivity with environmental non-pathogenic *Francisella* bacteria may occur [18]–[20]. Therefore there is a need to develop PCRs for distinguishing clinically relevant *Francisella* species from closely related non-pathogenic *Francisella* present in environmental sources.

In research laboratories, isolates of *F. tularensis* have been identified and classified using a variety of molecular typing methods, including amplified fragment length polymorphism (AFLP) analysis [21], pulse-field gel electrophoresis (PFGE) [22], [23], insertion/deletion (INDEL) mutation analysis [24], multi-locus variable number of tandem repeats analysis (MLVA) [25], [26], multi-locus sequence typing (MLST) [2], and whole genome single-nucleotide polymorphism (SNP) analysis [1]. The highest typing resolution has been achieved by MLVA of rapidly mutating tandem repeats, but at a cost sometimes of incorrectly characterizing relationships among distantly related isolates.

In the present study, we developed a convenient real-time PCR assay based on robust genetic markers (SNPs and INDELs). A desired feature of the assay was that it should be able to distinguish between human pathogenic *F.tularensis* and the two genetically closely related species *F. novicida* and *F. philomiragia* which are of lower clinical relevance and often found in environmental sources. Moreover, the assay should be capable of identifying the subclades of *F. tularensis* (especially within *F. tularensis* subsp. *holarctica*, type B), and be compatible with standard real-time PCR machines that are now widely used in routine diagnostic laboratories. The developed assay meets all of these criteria, and can be tailored to match typing resolution requirements by adding or removing genetic markers as appropriate.

## Materials and Methods

### Ethics Statement

Ulcer specimens were collected as part of the routine clinical management of patients and the use of them for laboratory service improvement conducted in compliance with the regulation, policies and principles of the Swedish Public Health Service. Approval from an ethics committee was for that reason not sought after. The clinical routine for collecting specimens includes an open friendly verbal communication informing the patient that the sampling purpose is detecting the causative agent of tularemia. A verbal informed consent was required before submitting any sample to the laboratory. The specimens were de-identified and analyzed anonymously.

### Isolates and Clinical Specimens

**Figure 2:**
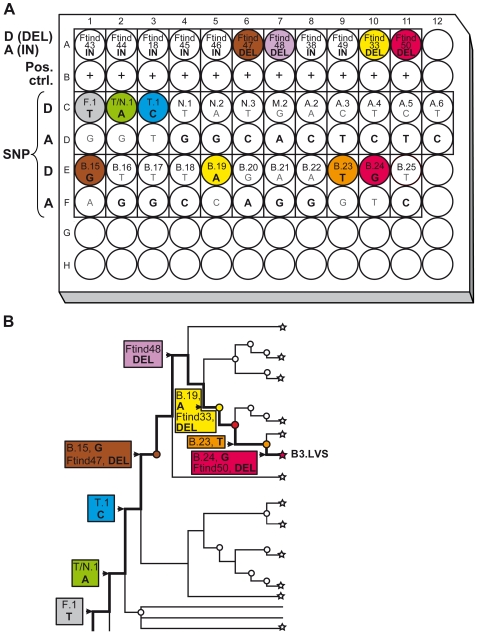
Example of plate design and interpretation of results for the genetic classification of *F. tularensis* strain LVS. A) The allelic state of each marker in the LVS strain is indicated in boldface. A colored well corresponds to a phylogenetically determining (canonical) marker for a specific genetic subclade. B) A phylogenetic tree is generated from hierarchical analysis of the typing results. Thick lines indicate the inferred evolutionary history of strain LVS. D = derived state, A = ancestral state.

A panel of 62 *Francisella* isolates (listed in Table 1), spanning as much as possible of the known genetic diversity within the genus, was used to determine the specificity of all of the tested markers (listed in Tables 2 and 3). The final one plate-assay, including 34 genetic markers, was applied to 14 isolates and six patient ulcer specimens obtained in 2008 at Umeå University Hospital, Sweden (Table 4), and also to five additional isolates of global origins (Table 1). The new assay was evaluated along with the standard PCR assay that is used for diagnosis of human ulceroglandular tularemia [27]. Plate design and interpretation of assay results are exemplified in Figure 2 by the analysis of the Live Vaccine Strain (LVS).

**Table 1:** Sixty-seven isolates of global origins used in this study.

Species (no. isolates)	origin	FSC no.^a^	NAU ID^b^	Alternative designations	Vogler *et al.* 2009 subclade	Johansson *et al.* 2004 group^c^	Table 5 geno-type	Figure 3 subclade
***F. philomiragia*** ** (5)**	Water, Bear River Refuge, UT	037	F0047	ATCC 25016	–	–	1	P.ATCC25017
	Water, Bear River Refuge, UT	038	F0048	ATCC 25017	–	–	1	P.ATCC25017
	Water, Odgen Bay Refuge, UT	039	F0049	ATCC 25018	–	–	1	P.ATCC25017
	Moribund muskrat (*Ondatra zibethicus*), 1959, Brigham City, UT	144	F0045	ATCC 25015	–	–	1	P.ATCC25017
	Atlantic cod (*Gadus morhua*), 2008, Norway	775^d^		DSM18777	–	–	1	P.ATCC25017
***F. novicida*** ** (5)**	Water, 1950, UT	040	F0050	ATCC 15482, U112	N	N	2	N.U112
	Human blood, 1991, Houston, TX	156^e^	F0051	fx1	N	N	3	N.FSC156
	Human blood, 1991, Houston, TX	159	F0052	fx2	N	N	3	N.FSC156
	Human blood, 2003, Spain	454		FNSp1, F62	–	–	4	N.FSC454
	Human, 2003, Brazil/UK/Germany	595		F58	–	–	5	N.Ftind44/[1], [2], [3]
***F. tularensis*** ** subsp. ** ***mediasiatica*** ** (4)**	Experimental isolate, cap-, Rostov, Russia	122	F0004	(TTC-R)6-4-1	M.Br.FSC 147	M	6	M.FSC147
	Midday gerbil (*Meriones meridianus*), 1965, Kazakhstan	147^e^	F0011	GIEM 543	M.Br.FSC 147	M	6	M.FSC147
	Hare, 1965, former USSR, Central Asia	149	F0013	120	M.Br.FSC 147	M	6	M.FSC147
	Tick, 1982, former USSR, Central Asia	148	F0012	240	M.Br.FSC 147	M	6	M.FSC147
***F. tularensis*** ** subsp. ** ***tularensis*** ** (11)**	1960 (Eigelsbach)	013	F0006	FAM standard	–	–	7	A1.3/[4], [5]
	Tick, 1935, British Columbia, Canada	041	F0005	Vavenby	A.I.Br.001/002	A1	7	A1.3/[4], [5]
	Squirrel, Georgia, USA	033^e^		SnMF	–	–	8	A1.FSC033
	Human pleural fluid, 1940, Fox Downs, Ohio, USA	046	F0008		A.I.Br.SCHU S4	A1	9	A1.SCHUS4
	Human, 1941, Ohio, USA	237	F0567	Schu S4	A.I.Br.SCHU S4	–	9	A1.SCHUS4
	Mites, 1988, Slovakia	199	F0007	SE-221/38	A.I.Br.SCHU S4	A1	9	A1.SCHUS4
	Lab acquired when handling Nevada 14	053	F0009	F.tul AC	A.II.Br.001/002	A2	10	A2
	Hare, 1953, Nevada, USA	054	F0010	Nevada 14	A.II.Br.001/002	A2	10	A2
	Hare, Canada	042	F0296	Utter	A.II.Br.003/004	A2	10	A2
	Human, 1920, Utah, USA	230	F0419	ATCC 6223	A.II.Br.ATCC 6223	A2	10	A2
	1959, USA	604		RKI 03-1300, 8859	–	–	10	A2
***F. tularensis*** ** subsp. ** ***holarctica*** ** (42)**	Human lymphnode, 1926, Japan	017	F0016	S-2	B.Br.001/002	B5	11	B5.FSC022
	Hare, 1954, Oniwa, Japan	020	F0292		–	B5	11	B5.FSC022
	Human, 1958, Tsuchiya, Japan	021	F0014		B.Br.001/002	B5	11	B5.FSC022
	Human, 1950, Ebina, Japan	022	F0015		B.Br.001/002	B5	11	B5.FSC022
	Tick, 1954, Fukushima, Japan	023	F0293	TH	–	B5	11	B5.FSC022
	Yerma, Japan	024	F0294		–	B5	11	B5.FSC022
	Tick, 1957, Jama, Japan	075	F0017		B.Br.001/002	B5	11	B5.FSC022
	Human blood, 1989, Norway	089	F0038	N1/89 (45F2)	B.Br.OSU18	B2	12	B2.OSU18
	Human blood, 1994, Bergen, Norway	158	F0301	CCUG 33391	B.Br.OSU18	B2	12	B2.OSU18
	Beaver, 1976, Montana, USA	035	F0018	B423A	B.Br.OSU18	B2	12	B2.OSU18
	Hare, 1997, Austria	584		F30	–	–	12	B2.OSU18
	Human ulcer, 2005, Ljusdal, Sweden	641^e^		05-32-85	–	–	12	B2.OSU18
	Human, 2000, Örebro, Sweden	285	F0212	AO7346/00	B.Br.007/008	B4	13	B4.Ftind49/18
	Tick, 1941, Montana, USA	012	F0291	425 F4G	–	–	13	B4.Ftind49/18
	Human ulcer, 2004, Örebro, Sweden	519		04-32-23	–	–	13	B4.Ftind49/18
	Human, 2004, Umeå, Sweden	663^d^			–	–	13	B4.Ftind49/18
	Human, 2000, Uppsala, Sweden	274	F0228	R63/00	B.Br.010/011	Spain, France, & Sweden	13	B4.Ftind49/18
	Human, 1993/94, Vosges, France	247	F0020	T 20	B.Br.FTNF002-00	Spain, France, & Sweden	14	B4.FTNF002-00
	Hare, 1952, Chateauroux, France	025	F0295	061-1	B.Br.FTNF002-00	Spain, France, & Sweden	14	B4.FTNF002-00
	Hare, Castilla y León, Spain	455		FT1	–	–	14	B4.FTNF002-00
	Human skin lesion, Castilla y León, Spain	456		FT7	–	–	14	B4.FTNF002-00
	Human, 1995, Ockelbo, Sweden	162	F0162		B.Br.012/013	B3	15	B3.19/[20], [23]
	Human, 1995, Ockelbo, Sweden	178	F0044		B.Br.012/013	B3	15	B3.19/[20], [23]
	Water, 1980, Crimea, Ukraine	115	F0021		B.Br.013/014	B3	15	B3.19/[20], [23]
	Norwegian rat (*Rattus norvegicus*), 1988, Rostov, Russia	150	F0029		–	B3	15	B3.19/[20], [23]
	Human blood, 1996, Raahe, Finland	250	F0164		B.Br.013/014	B3	16	B3.23/[24], [25]
	Human lymph node, 2005, Summi Admin area, Ukraine	FDC079^d^			–	–	16	B3.23/[24], [25]
	Live vaccine strain, Russia	155	F0566		B.Br.LVS	B3	17	B3.LVS
	Tick (*Dermacentor pictus*), 1949, Moscov area, Russia	257e,f	F0019	GIEM 503/840	B.Br.013/014	B3	18	B3.RC503
	Tick (*Dermacentor reticularis*), 1995, Lanzhot, Czech Republic	184^d^	F0191	T-35	–	B1	19	B1.20/21
	Tick (*Dermacentor reticularis*), 1995, Lanzhot, Czech Republic	185	F0192	T-38	B.Br.013/014	B1	19	B1.20/21
	Tick (*Dermacentor reticularis*), 1995, Lanzhot, Czech Republic	186	F0193	T-44	B.Br.013/014	B3	19	B1.20/21
	Tick (*Ixodes ricinus*), 1995, Lanzhot, Czech Republic	187	F0194	T-60	B.Br.013/014	B1	19	B1.20/21
	Bank vole (*Clethrionomys glareolus*), 1977, Seneca district, Slovakia	FDC010			–	–	19	B1.20/21
	Brown hare (*Lepus europaeus*), 1964, Vidiek district, Slovakia	FDC014			–	–	19	B1.20/21
	Water, 1985, Rostov region, Russia	121	F0025	12267	B.Br.013/014	B1	19	B1.20/21
	Human, 1995, Äänekoski, Finland	249	F0163	1468	B.Br.013/014	B1	19	B1.20/21
	Water, 1990, Odessa region, Ukraine	124	F0027	14588	B.Br.013/014	B1	20	B1.21/22
	Water, 1990, Odessa region, Ukraine	119^d^		14592	–	B1	20	B1.21/22
	Human, 2001, Oulu university hospital, Finland	293	F0178	T-10023	B.Br.013/014	–	20	B1.21/22
	Human, 1998, Ljusdal, Sweden	200	F0134	3001MA	B.Br.013/014	B1	21	B1.FSC200
	Human ulcer, 1995, Ljusdal, Sweden	245	F0133	R42/95	B.Br.013/014	B1	21	B1.FSC200

a Strain ID in the *Francisella* Strain Collection (FSC) and *Francisella* DNA Collection (FDC), Swedish Defense Research Agency, Umeå, Sweden.

b Strain ID in the Northern Arizona University DNA collection.

c MLVA-defined groups presented in Johansson et al. 2004. A1, *F. tularensis* subsp. *tularensis* subpopulation A1; A2, *F. tularensis* subsp. *tularensis* subpopulation A2; B, *F. tularensis* subsp. *holarctica*; M, *F. tularensis* subsp. *mediasiatica*; N, *F. novicida*.

d The isolates FDC079, FSC119, FSC184, FSC663, and FSC775 (*F. philomiragia* subsp. *noatunensis*) were typed with the final one-plate assay and were not part of the set of 62 isolates used in the developing stage.

e The isolates FSC017 (B5), FSC033 (A1), FSC147 (M), FSC156 (N), FSC257 (B3), and FSC641 (B2) were typed with the final one-plate assay, and were part of the set of 62 isolates used in the developing stage, to confirm the typing accuracy of the plate.

f FSC257 is an alternative name for RC503.

**Table 2:** SNP markers, genes affected by the SNPs, and primers.

SNP	SCHU S4^a^ SNP position	SCHU S4 locus ID	SCHU S4 gene	SNP state	Primer^b^	Primer sequences^c^
**F.1**	1312210,	FTTr04,	16S	T	D	gcgggcCTATGGATCGTAGCCTTGGt
	1379332,	FTTr10,		G^d^	A	gcgggcagggcggcCTATGGATCGTAGCCTTGGg
	1772676	FTTr07			C	AGTTGGAAACGACTGTTAATACCGCA
**T/N.1**	83976	FTT0080	*tpiA*	A	D	gcgggcAGAAACACATCAATTTATTCGTTCa
				G	A	gcgggcagggcggcAGAAACACATCAATTTATTCGTTCg
					C	AGCATTTTCAGCTTTTAGGCTACCA
**T.1**	1165690	FTT1150c	*putA*	C	D	gcgggcagggcggcTGTTGAAAAAGCTCATATGTCAAGc
				T	A	gcgggcTGTTGAAAAAGCTCATATGTCAAGt
					C	TCATACTCGATCATAAACGCATCA
**N.1**	83943	FTT0080	*tpiA*	T	D	gcgggcACAGGAGTTGTGGCTTCACTAGAt
				G	A	gcgggcagggcggcACAGGAGTTGTGGCTTCACTAGAg
					C	CATCAACTTTAGCTAACAATGAACGAAT
**N.2**	910194	FTT0901	*lpnA*	A	D	gcgggcTGTAATCTTACACTTCCTTGTGGa
				G	A	gcgggcagggcggcTGTAATCTTACACTTCCTTGTGGg
					C	GGCTCTGATGATGCAAAAGC
**N.3**	780	FTT0001	*dnaA*	T	D	gcgggcGCAGATCTATAAACTCTTTGAAAt
				C	A	gcgggcagggcggcGCAGATCTATAAACTCTTTGAAAc
					C	AATTTATTAAAGATTATGTAAATTCTATTCGT
**M.2**	84027	FTT0080	*tpiA*	G	D	gcgggcagggcggcTCAGCTTTTAGGCTACCACCg
				A	A	gcgggcTCAGCTTTTAGGCTACCACCa
					C	CAGGAGTTGTGGCTTCACTAGAGC
**A.2**	1199395	FTT1182c	*vacJ*	A	D	gcgggcGCATCAACACTATCACTAATCCCCTa
				C	A	gcgggcagggcggcGCATCAACACTATCACTAATCCCCTc
					C	ATCACCAAGATTTTGCTGTGACATT
**A.3**	62997	FTT0062	*atpA*	C	D	gcgggcagggcggcTGCTGTAGCTGCAACAATAATTGc
				T	A	gcgggcTGCTGTAGCTGCAACAATAATTGt
					C	ATTGCAAACATTGTAAGACAGCTTGAAG
**A.4**	830716	FTT0810	*ybaB*	T	D	gcgggcTCGGTAAGTATCGACAATTt
				C	A	gcgggcagggcggcTCGGTAAGTATCGACAATTc
					C	AGCAGCTGCTATCAAATCTTC
**A.5**	350750	FTT0351	*rplQ*	C	D	gcgggcagggcggcTAGAGGCTCAACGATTGc
				T	A	gcgggcTAGAGGCTCAACGATTGt
					C	TGTCAGCTTCTTTGATTAATC
**A.6**	1806912	FTT1721	*purF*	T	D	gcgggcTCGTACTCTTTAAAACCAAGCAt
				C	A	gcgggcagggcggcTCGTACTCTTTAAAACCAAGCAc
					C	CTGAGGCTGTTTATAAAGCATGTAAAT
**B.15**	1113816	FTT1103		G	D	gcgggcagggcggcTCAACTTGGAATCCAAGGCg
				A	A	gcgggcTCAACTTGGAATCCAAGGCa
					C	GCTTTGTTGATAGCTGCTTGGATACC
**B.16**	608246	FTT0588	*aroA*	T	D	gcgggcATGCTAGCAAATTACCATCAAAAGt
				G	A	gcgggcagggcggcATGCTAGCAAATTACCATCAAAAGg
					C	AACTCTTCTCGCCATCAACTTCTAT
**B.17**	1743251	FTT1673	*ribA*	T	D	gcgggcCCAAGAGCTAAATTAGCTTCAAt
				G	A	gcgggcagggcggcCCAAGAGCTAAATTAGCTTCAAg
					C	TGACCAAGAAGGTAGAGGTATTGGTT
**B.18**	1756146	FTT1686c		T	D	gcgggcAGCAGCAGGACAAATAGt
				C	A	gcgggcagggcggcAGCAGCAGGACAAATAGc
					C	TTGTGTCGATTCAAAACCAGACTTA
**B.19**	1374034	FTT1343c		A	D	gcgggcTTGCTACTGATGGTTTAACTa
				C	A	gcgggcagggcggcTTGCTACTGATGGTTTAACTc
					C	CAATACGTCACTTATGCAGTGAT
**B.20**	1396117,	FTT1354,	*pdpC*	G	D	gcgggcagggcggcTCTGATGAAGAATATCTTACAg
	1789461	FTT1709		A	A	gcgggcTCTGATGAAGAATATCTTACAa
					C	ATTATGGCAAAACTATACCTT
**B.21^e^**	701320	FTT0684c	*sthA*	A	D	gcgggcACCAAGGTAGATTTGCAGCTACa
				G	A	gcgggcagggcggcACCAAGGTAGATTTGCAGCTACg
					C	ATCCCTGTTGGGATATCCTCGACTAA
**B.22^e^**	1113320	FTT1103		A	D	gcgggcTGAATACTCTACGCGATAAGATa
				G	A	gcgggcagggcggcTGAATACTCTACGCGATAAGATg
					C	ATCAGACTTAGGTGTTAGATCAGAGTT
**B.23**	253121	FTT0240		T	D	gcgggcTTACTACAAATTCGCCTCTAAt
				G	A	gcgggcagggcggcTTACTACAAATTCGCCTCTAAg
					C	AGCAAAAGAGCTTACTAAACAATTTGA
**B.24**	1419996	FTT1373	*fabH*	G	D	gcgggcagggcggcTATCGCCAGGTTTAATTTGATg
				T	A	gcgggcTATCGCCAGGTTTAATTTGATt
					C	TCTGCAGCATCTATCCCATTAGCCTTA
**B.25**	1534495	FTT1484c	*aceF*	T	D	gcgggcTGTATCTAAGACAGCAGTGAAGt
				C	A	gcgggcagggcggcTGTATCTAAGACAGCAGTGAAGc
					C	ATGGTAGCATAGTTCTAGGAATAAACT

a GenBank accession no. AJ749949.

b D, Primer with derived SNP state; A, Primer with ancestral SNP state; C, Common primer.

c Primer tails and 3′-end mismatch base are in lower case.

d No sequences with a G found by BLAST search against the nt database 2009/22/04, among isolates of the family of *Francisellacae*, uncultured and environmental *Francisella*-like bacteria.

e B.21 is identical to Ft-SNP1 and B.22 is identical to Ft-SNP2 in Svensson et al 2009 (submitted manuscript).

**Table 3:** INDEL markers, genes affected by the INDELs, and primers.

INDEL	SCHU S4 INDEL position	SCHU S4 locus ID	SCHU S4 gene	Primer	Primer sequences
**Ftind43**	1541234..1541239	FTTt30, FTTt31	Arg-tRNA, Gly-tRNA	IN	GTTTCACAAATTTGCGGGAA
			(intergenic)	OUT	AATCCCTTTGGGTGTGCCAT
				CP	TGGAGCGGGAAACGAGGC
**Ftind44**	895956..896021	FTT0886, FTT0887	*recN*, FTT0887	IN	TCGACAAGTAGTTACTCAGCCTA
			(intergenic)	OUT	TAAATCTAGTTGGCTGAGTAAT
				CP	ACTGTTGTCATTCCCACGTA
**Ftind18^b^**	439349..439371	FTT0425	*asd*	IN	AGACCCTCTAAATCACGATCA
				OUT	AGGTTTCTGGATAGACGCTGCA
				CP	ACTAACAGTACAATTACTACCGAT
**Ftind45**	725227..725228	FTT0706	*glk2*	IN	ACCTAATATGACCATAGATGGAT
			(pseudogene)	OUT	TCACCAATAGCTTCCATAACA
				CP	ACTCAGTGAAGCTATGGAATATCT
**Ftind46**	1830698..1830699	FTT1739	*kdpA*	IN	AGTTCTGTACTGCAAGAGCGA
			(pseudogene)	OUT	GTAGCTGTTTCATGCCTTGCT
				CP	AGCACTTAATACAGCAGTTAGT
**Ftind47**	271674..271683	FTT0255		IN	AGTAATACGCAAAGATTTTCTACA
				OUT	TCTTAACTGTATGCTAGTCTATGA
				CP	TAATAGAGCGGCTCTTCGAAT
**Ftind48**	960987..961011	FTT0948		IN	ATCCTACTAATATCAATTCCAGT
				OUT	CCTTCAGCTTGAGTATTTTGACGT
				CP	ACTGTTATATTCAGTTATTTGCT
**Ftind38** b	95661..95674	FTT0092	*appC*	IN	ACCCAATAAGCTCACCATCA
			(pseudogene)	OUT	ATCTTTCTCAGGTACAGACTTTA
				CP	AGTACTATTTGCTTATCCAAGTGAA
**Ftind49**	834341..834349	FTT0816		IN	AAGATTAAGTGGCAATTTAC
				OUT	TTCAACCTGGACAACCACTA
				CP	AGGATCCCAGTTAGGTTTAGTA
**Ftind33^b^**	512045..512063	FTT0492	*lysR*	IN	TCTAAATTTAAGCAATGTTTCTAACT
				OUT	ATCATCGTATAAGAAATCAACTT
				CP	TCAACCTTACAGAATAAGAATGT
**Ftind50**	88484..88576	FTT0086		IN	CATCACTGCCACCAAGCATAT
				OUT	TGGGCACCATAAATAGCTAGT
				CP	CGATGCCATGGTCAGATGATCA

a GenBank accession no. AJ749949.

b Ftind18, Ftind33 and Ftind38 were previously used in [24].

**Table 4:** Fourteen isolates and six ulcer specimens from tularemia patients in Sweden 2008 characterized by the developed hierarchical real-time PCR array.

Category	FSC no.^a^	Sample ID^b^	Location of the receiving hospital	Table 5 genotype	Figure 3 subclade
**Isolates**	792	32–92	Säffle	13	B4.Ftind49/18
	844	32–280	Uddevalla	13	B4.Ftind49/18
	780	32–51	Luleå	16	B3.23/[24], [25]
	785	32–75	Falun	16	B3.23/[24], [25]
	812	32–123	Sunderbyn	16	B3.23/[24], [25]
	816	32–142	Boden	16	B3.23/[24], [25]
	823	32–155	Lövånger	16	B3.23/[24], [25]
	831	32–173	Skellefteå	16	B3.23/[24], [25]
	794	24–95	Östersund	19	B1.20/21
	777	32–38^c^	Örebro	19	B1.21/22
	787	32–79	Umeå	20^d^	B1.21/22
	778	32–47^c^	Ljusdal	20^d^	B1.21/22
	783	32–69	Färila	21	B1.FSC200
	817	32–145	Bollnäs	21	B1.FSC200
**Ulcer specimens**	–	32–151^e^	Jönköping^f^	16, 17 or 18^g^	B3.23/[24], [25]
	–	32–300^e^	Gävle	16, 17 or 18^g^	B3.23/[24], [25]
	–	32–87^e^	Umeå	16, 17 or 18^g^	B3.23/[24], [25]
	–	32–215 e	Uddevalla	19^h^	B1.20/21
	–	32–38^c^	Örebro	20^h^	B1.21/22
	–	32–47^c^	Ljusdal	20^h^	B1.21/22

a Strain ID in the *Francisella* Strain Collection, Swedish Defense Research Agency, Umeå, Sweden.

b Sample ID at the Department of Clinical Bacteriology, Umeå University, Umeå, Sweden.

c Isolate FSC777 and ulcer specimens 32–38 are from the same patient. Isolate FSC778 and ulcer specimens 32–47 are from the same patient.

d The exact genotype could not be determined due to detection failure of marker B.22 (the difference in time of appearance between the two PCR products was less than one cycle).

e *F. tularensis* cultures were negative.

f The patient reported probable acquisition of tularemia when visiting the county of Jämtland, where the regional center is Östersund.

g The genotype and subclade were assigned based on marker B.20, which exhibited an A for all three specimens, and on marker B.23, which exhibited a T. No other markers were screened due to scarcity of DNA.

h The genotype and subclade were assigned based on: marker B.20, which exhibited a G for all three specimens; on marker B.21, which exhibited a G for specimens 32–215, and an A for specimens 32–38 and 32–47; and on marker B.22, which exhibited a G for specimens 32–38 and 32–47. No other markers were screened due to scarcity of DNA.

### DNA Preparation

*F. tularensis* isolates were re-cultured and a loopful of each isolate was suspended in phosphate buffered saline, heat-killed and DNA was prepared by phenol/chloroform extraction using Phase Lock Gel Light tubes (Eppendorf, Hamburg, Germany) or by a chaotropic salt method [27]. The latter was also used to prepare DNA from the clinical specimens. The concentration of DNA in each sample was determined using a BioPhotometer (Eppendorf, Hamburg, Germany) or NanoDrop spectrophotometer (Thermo Scientific, Wilmington, DE, USA), then adjusted to 2.5 ng/µl.

### Genetic Markers and Primers

Phylogenetically informative SNPs and INDELs were identified by BLAST searches of available *Francisella* genomes and DNA sequences at the National Centre for Biotechnology Information (NCBI). In addition, two INDELs and 12 SNPsC previously shown to discriminate between isolates of *Francisella* were selected and tested for specificity [2], [24], [28]–[30].

For each SNP marker, two forward allele-specific primers with different 3′ bases, each matching one of the SNP allele states, and a reverse common primer, were designed using Primer3 [31] (Table 2). The primers were designed according to a SNP discrimination assay described by Germer and Higuchi [32], [33], in which GC-rich tails of different lengths are added to each of the two allele-specific primers: a 14 bp (GCGGGCAGGGCGGC) tail was attached to the primer with G or C at the 3′-end, and a six bp (GCGGGC) tail was attached to the primer with A or T at the 3′-end. The GC-tails were in the original publication added primary to obtain a difference in the melting temperature, but a larger difference in the time of appearance between the two PCR-products was also obtained. For each INDEL marker, one common primer (CP) and two forward primers were designed: one inside (IN) and one outside (OUT) the deletion (Table 3). The CP-OUT primer pair was used as a positive control.

All primers were obtained (from Eurofins MWG, Ebersberg, Germany) and matched regions with an identical nucleic acid sequence in compared genomes and DNA sequences of the genus *Francisella* to minimize amplification failure of screened isolates.

### Real-time PCR

In the final assay, real-time PCR amplifications of 34 genetic markers were performed using an iCycler (BioRad) with 5 ng DNA, or a Mastercycler instrument (Eppendorf) with 2 ng DNA, in both cases in 25 µl reaction mixtures in 68 wells of a 96-well plate (one primer pair per well was used). The reaction mixture for SNP detection consisted of 5 pmol of each primer (MWG-Biotech), 3U of AmpliTaq DNA Polymerase Stoffel Fragment, 2 mM MgClB_2B_, 50 µM dNTP, 20x SYBR Green I, 4% dimethyl sulfoxide (DMSO), and 2% glycerol. Two master mixes were prepared in which each of the allele-specific primers were added. The amplification conditions were: 50°C for 2 min, 95°C for 10 min, followed by 40 cycles at 95°C for 15 sec and 60°C for 1 min. The SNP in each sample was determined by inspecting the amplification curves. Amplification appeared earlier in reaction mixtures containing the forward primers with a matching 3′-base. A positive result was assigned when there was a one cycle or more difference between the time of appearance of PCR-products, and the number of cycles did not exceed 35. For INDEL analysis, Power SYBR Green PCR Mastermix was used with the same cycling conditions as for SNPs. The presence of a deletion was detected by failure of the reaction mixture with one primer in the deleted sequence to yield a detectable amplification product, while the control reaction with primer pairs surrounding the deletion succeeded. For cases where one primer overlapped a small deletion a minimum detection threshold of a five-cycle difference in time of appearance between the control and test reactions was set.

### Quality Controls

The final 68-well assay included one PCR reaction per well (no multiplexing). To evaluate the typing accuracy of the assay, a test blinded to the investigator was performed on a subset of six isolates previously used in the development stage and representing the MLVA genetic groups *F. novicida* (N), *F. t. mediasiatica* (M), A1, B2, B3, and B5 of *Francisella*. Genetic group designations are found in Figure 1 and Table 1. The detection limit of the final assay was tested with serial logarithmic dilutions of *F. tularensis* subsp. *holarctica* Live Vaccine Strain (LVS) DNA, starting at one ng. The detection limit was set at the lowest amount of DNA with which PCR amplification of all 34 markers occurred. The reproducibility of the assay was assessed using one ng DNA of LVS tested in three replicate runs.

### MLVA

To assign MLVA clusters for isolates that had not been previously characterized in [26], MLVA was performed using a CEQ 8800 instrument (Beckman Coulters, Fullerton, CA), as previously described [26].

### Accession Numbers

Completed genomic sequences (with GenBank accession numbers in parenthesis) used in this work were: U112 (CP000439), ATCC25017 (CP000937), WY96-3418 (CP000608), FSC147 (CP000915), FTNF002-00/FTA (CP000803), OSU18 (CP000437), LVS (AM233362) and SCHUS4 (AJ749949).

Draft genome sequences (with GenBank accession numbers in parenthesis) used in this work were: ATCC25015 (ABYY00000000), FSC200 (AASP00000000), FTE (ABSS00000000) and FTG (ABXZ00000000).

Preliminary sequence data were obtained from the MIT Broad Institute website at www.broad.mit.edu for the following *Francisella* strains: GA99-3549, GA99-3548, FSC033, FSC022, and FSC257/RC503.

The following *Francisella* genomes from Baylor College of Medicine Human Genome Sequencing Center website at www.hgsc.bcm.tmc.edu were not available at the time of the study, but are mentioned here: ATCC6223, KO97-1026, MI00-1730 and OR96-0246/BSA; The OR96-0463 genome was sequenced by the Joint Genome Institute and Lawrence Livermore National Laboratory, and is available from http://genome.ornl.gov.

The following previously published genes found to discriminate between isolates of *Francisella*, were used: *dnaA* (AM261088 to AM261101) [29]; *tpiA* (AM261102 to AM261115 [29], AY794514 to AY794528 and AY794497 [2]); *lpnA/tul4/17kD* (AM261150 to AM261161 and AM261164) [29]; *putA* (AM261165 to AM261178) [29]; *aroA* (AY794435 to AY794449 and AY794495) [2]; *atpA* (AY794498 to AY794513) [2]; *vacJ* (DQ451123 to DQ451126) [28]; *fabH* (DQ863407 to DQ863420) [30]; FTT0086 (DQ863472 to DQ863483) [30]; *asd*/FTT0425 (Ftind18) and *lysR*/FTT0492 (Ftind33) [24]; *appC*/FTT0092 (Ftind38) [24], and *aceF*, RD17 (AY794422) [2].

## Results

### Selection of Genetic Markers

We identified 49 SNPs and 15 INDELs with potential canonical properties by analyzing various available DNA sequences. Strain polymorphism was verified using a pair of isolates showing the two possible allelic states. Twenty-four SNPs and three INDELs were not used in further analyses because of amplification failure, or (in SNP analysis) because there was a less than one cycle difference in the time of appearance of different PCR products. In evaluation of the remaining SNPs and INDELs in a panel of 62 *Francisella* isolates of diverse genetic and geographical origins, two SNPs and one INDEL were found to be incongruent with the phylogenetic structure of *F. tularensis* determined by Vogler et al [1], and were therefore also discarded. The final set of markers comprised 23 SNPs and 11 INDELs, which were arrayed in a hierarchical assay structure in 68 wells of a 96-well plate (one primer pair per well was used) (Figure 2).

### Detection Level and Typing Resolution

**Figure 3:**
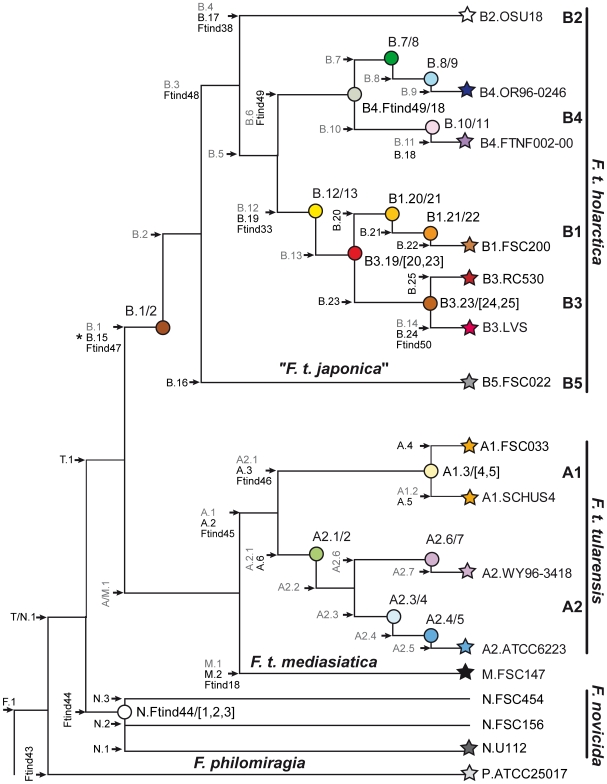
Schematic SNP and INDEL phylogeny, indicating genetic markers and *Francisella* subclades. Markers presented in this study are indicated in black and, for comparison, SNP markers developed in a recent study by Vogler et al 2009 [1] are indicated in gray. The branch names of Vogler et al. have been abbreviated to simplify the nomenclature. Stars indicate terminal subclades defined by *Francisella* genomes and circles represent collapsed branch points along the genetic lineages that contain isolates of a particular genotype (a subclade). The subclades are named for the flanking SNPs and INDELs. The branch lengths do not represent true phylogenetic distances. The position of B.15/Ftind47 (marked by the asterisk in the figure) could not be definitively determined; it could be either where shown, or be descendant from B.1/2.

The limit of detection of our assay was found to be 100 pg DNA. Three replicate runs using *F. tularensis* strain LVS showed identical results. An indefinite typing result occurred on average in 0.3 to one marker per plate. However, unambiguous strain classification was still possible using the information obtained from the other markers. The assay successfully detected and discriminated among the three species of *Francisella*, the five major genetic clades of *F. tularensis,* and the subclades of *F. tularensis* subsp. *holarctica*. A comparison with a set of recently published canonical SNPs [1] showed perfect correlation with the results obtained in our assay (as shown in the *Francisella* phylogeny depicted in Figure 3, which indicates names of markers and subclades from both research groups). Our markers B.20 to B.23 and B.25, B.16 and A.4 added typing resolution to the genetic branches B.Br.013/014, B.Br.002/003, and A.I.001/002 previously defined by Vogler et al [1] (Figure 3, Table 5). In addition, the use of INDELs Ftind44, Ftind48 and Ftind49 provided resolution at phylogenetic nodes where no corresponding SNP was identified by Vogler et al. (Figure 3). Our markers T.1 and Ftind44 also conveniently discriminated all *F. tularensis* strains from *F. novicida*, *F. philomiragia* and *F. noatunensis* isolates (Figure 3, Table 5).

**Table 5:** *Francisella* genotypes in this study.

Genotype	F.1	Ftind 43	T/N.1	T.1	Ftind 44	N.1	N.2	N.3	Ftind 18^a^	M.2	Ftind 45	A.2	Ftind 46	A.3	A.4	A.5	A.6	Ftind 47	B.15	Ftind 48	B.16	Ftind 38^a^	B.17	Ftind 49	B.18	Ftind 33	B.19	B.20	B.21	B.22	B.23	Ftind 50	B.24	B.25
**1. P.ATCC25017**	**T** b	**DEL** c	G	T	ND^d^	ND	ND	ND	ND	ND	ND	ND	ND	ND	ND	ND	ND	ND	ND	ND	ND	ND	ND	ND	ND	ND	ND	ND	ND	ND	ND	ND	ND	ND
**2. N.U112**	**T**	IN^e^	**A**	T	**DEL**	**T**	G	C	IN	A	IN	C	IN	T	C	T	C	IN	A	IN	G	IN	G	IN	C	IN	C	A	G	G	G	IN	T	C
**3. N.FSC156**	**T**	IN	**A**	T	**DEL**	G	**A**	C	IN	A	IN	C	IN	T	C	T	C	IN	A	IN	G	IN	G	IN	C	IN	C	A	G	G	G	IN	T	C
**4. N.FSC454**	**T**	IN	**A**	T	ND	G	G	**T**	IN	A	ND	C	ND	T	C	T	C	IN	A	IN	ND	ND	G	ND	C	ND	ND	A	G	G	G	ND	T	T
**5. N.Ftind44/** **[1], [2], [3]**	**T**	IN	**A**	T	**DEL**	G	G	C	IN	A	IN	C	IN	T	C	T	C	IN	A	IN	G	IN	G	IN	C	IN	C	A	G	G	G	IN	T	C
**6. M.FSC147**	**T**	IN	**A**	**C**	IN	G	G	C	**DEL**	**G**	IN	C	IN	T	C	T	C	IN	A	IN	G	IN	G	IN	C	IN	C	A	G	G	G	IN	T	C
**7. A1.3/** **[4], [5]**	**T**	IN	**A**	**C**	IN	G	G	C	IN	A	**DEL**	**A**	**DEL**	**C**	C	T	C	IN	A	IN	G	IN	G	IN	C	IN	C	A	G	G	G	IN	T	C
**8. A1.FSC033**	**T**	IN	**A**	**C**	IN	G	G	C	IN	A	**DEL**	**A**	**DEL**	**C**	**T**	T	C	IN	A	IN	G	IN	G	IN	C	IN	C	A	G	G	G	IN	T	C
**9. A1.SCHUS4**	**T**	IN	**A**	**C**	IN	G	G	C	IN	A	**DEL**	**A**	**DEL**	**C**	C	**C**	C	IN	A	IN	G	IN	G	IN	C	IN	C	A	G	G	G	IN	T	C
**10. A2.1/2**	**T**	IN	**A**	**C**	IN	G	G	C	IN	A	**DEL**	**A**	IN	T	C	T	**T**	IN	A	IN	G	IN	G	IN	C	IN	C	A	G	G	G	IN	T	C
**11. B5.FSC022**	**T**	IN	**A**	**C**	IN	G	G	C	IN	A	IN	C	IN	T	C	T	C	**DEL**	**G**	IN	**T**	IN	G	IN	C	IN	C	A	G	G	G	IN	T	C
**12. B2.OSU18**	**T**	IN	**A**	**C**	IN	G	G	C	IN	A	IN	C	IN	T	C	T	C	**DEL**	**G**	**DEL**	G	**DEL**	**T**	IN	C	IN	C	A	G	G	G	IN	T	C
**13. B4.Ftind49/18**	**T**	IN	**A**	**C**	IN	G	G	C	IN	A	IN	C	IN	T	C	T	C	**DEL**	**G**	**DEL**	G	IN	G	**DEL**	C	IN	C	A	G	G	G	IN	T	C
**14. B4.FTNF002-00**	**T**	IN	**A**	**C**	IN	G	G	C	IN	A	IN	C	IN	T	C	T	C	**DEL**	**G**	**DEL**	G	IN	G	**DEL**	**T**	IN	C	A	G	G	G	IN	T	C
**15. B3.19/** **[20], [23]**	**T**	IN	**A**	**C**	IN	G	G	C	IN	A	IN	C	IN	T	C	T	C	**DEL**	**G**	**DEL**	G	IN	G	IN	C	**DEL**	**A**	A	G	G	G	IN	T	C
**16. B3.23/** **[24], [25]**	**T**	IN	**A**	**C**	IN	G	G	C	IN	A	IN	C	IN	T	C	T	C	**DEL**	**G**	**DEL**	G	IN	G	IN	C	**DEL**	**A**	A	G	G	**T**	IN	T	C
**17. B3.LVS**	**T**	IN	**A**	**C**	IN	G	G	C	IN	A	IN	C	IN	T	C	T	C	**DEL**	**G**	**DEL**	G	IN	G	IN	C	**DEL**	**A**	A	G	G	**T**	**DEL**	**G**	C
**18. B3.RC530**	**T**	IN	**A**	**C**	IN	G	G	C	IN	A	IN	C	IN	T	C	T	C	**DEL**	**G**	**DEL**	G	IN	G	IN	C	**DEL**	**A**	A	G	G	**T**	IN	T	**T**
**19. B1.20/21**	**T**	IN	**A**	**C**	IN	G	G	C	IN	A	IN	C	IN	T	C	T	C	**DEL**	**G**	**DEL**	G	IN	G	IN	C	**DEL**	**A**	**G**	G	G	G	IN	T	C
**20. B1.21/22**	**T**	IN	**A**	**C**	IN	G	G	C	IN	A	IN	C	IN	T	C	T	C	**DEL**	**G**	**DEL**	G	IN	G	IN	C	**DEL**	**A**	**G**	**A**	G	G	IN	T	C
**21. B1.FSC200**	**T**	IN	**A**	**C**	IN	G	G	C	IN	A	IN	C	IN	T	C	T	C	**DEL**	**G**	**DEL**	G	IN	G	IN	C	**DEL**	**A**	**G**	**A**	**A**	G	IN	T	C

a Ftind18, Ftind33 and Ftind38 were previously used in [24].

b A boldfaced marker corresponds to a phylogenetically determining (canonical) marker for a specific genetic subclade.

c DEL = derived deletion.

d ND = not detected.

e IN = ancestral state.

### Concordance to MLVA

The categorization of *F. tularensis* isolates based on 23 SNPs and 11 INDELs was consistent with the MLVA-groupings presented by Johansson et al in 2004 [26] (Table 1, Figure 1) with one exception. In our SNP/INDEL analysis, strain FSC186 was classified as belonging to B1, while it was classified as B3 by MLVA [26]. An analysis of MLVA data showed that the inconsistency was likely caused by homoplasy (characters shared by a set of strains but not present in their common ancestor) at the highly variable MLVA markers Ft-M3 and Ft-M6 (Table 6).

**Table 6:** Repeat numbers for isolates within subclades B1 and B3 of *F. tularensis* subsp. *holarctica* at four MLVA-loci.

Isolate ID	Johansson *et al.* 2004 group	In this study	Ft-M3	Ft-M6	Ft-M20	Ft-M21
**FSC162**	B3	B3	17	4	3	2
**FSC178**	B3	B3	17	4	3	2
**FSC115**	B3	B3	13	4	3	3
**FSC150**	B3	B3	14	4	3	2
**FSC250**	B3	B3	21	4	3	2
**FSC155**	B3	B3	16	4	4	2
**FSC257**	B3	B3	17	4	3	4
**FSC185**	B1	B1	11	5	3	2
**FSC186**	B3	B1	12	4	3	2
**FSC187**	B1	B1	12	6	3	2
**FDC010**	–	B1	10	4	3	2
**FDC014**	–	B1	10	6	3	2
**FSC121**	B1	B1	9	6	3	2
**FSC249**	B1	B1	9	6	4	2
**FSC124**	B1	B1	17	6	3	2
**FSC293**	–	B1	17	5	3	2
**FSC200**	B1	B1	10	5	3	2
**FSC245**	B1	B1	10	5	3	2

### Categorization of *Francisella* Strains by the Real-time PCR array

**Figure 4:**
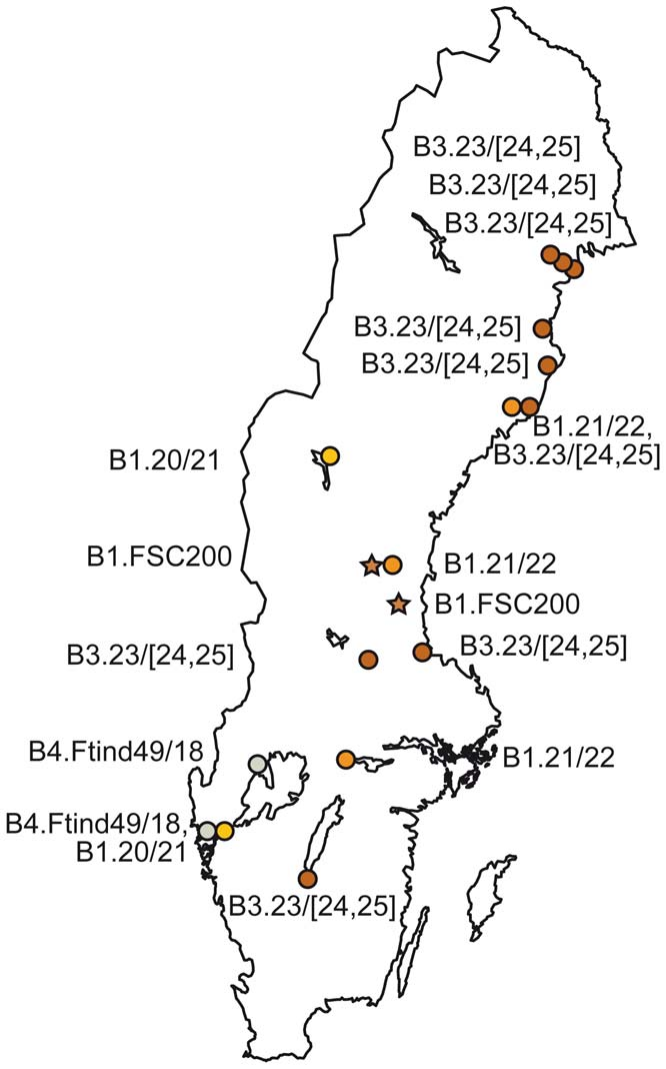
Example of use. The subclade names for 14 isolates and six ulcer specimens from tularemia patients in Sweden 2008 (Table 4) genotyped by the developed hierarchical real-time PCR array, and the location of the receiving hospitals.

Twenty-one genotypes were detected by the hierarchical array (Table 5). The typing accuracy of the final one-plate assay was assessed in a blind test, in which we correctly categorized six isolates previously tested individually for each marker. We further used the assay to categorize 14 isolates obtained from patients with tularemia in Sweden in 2008 (Figure 4, Table 4), and five isolates that were not included in the development of the assay (Table 1). We characterized six human tularemia ulcer specimens that were positive by the standard PCR for diagnosis of ulceroglandular tularemia [27] by amplifying the four selected markers B.20 to B.23 (Figure 4, Table 4), since we could not apply the new assay with all 34 markers due to scarcity of DNA.

## Discussion

In the present study we combined analysis of INDELs and SNPs in a real-time PCR array for robust, rapid and flexible hierarchical identification of *F. novicida* and *F. philomiragia*, and typing of human pathogenic members of the genus *Francisella*. In contrast to previously published real-time PCR assays, our assay was designed to cover the full currently known phylogenetic range of *Francisella*. The assay was also tailored to provide high typing resolution for *F. tularensis* subsp. *holarctica* isolates originating from Scandinavia, where our laboratory is located. Hierarchical typing based on cultivation and bacterial phenotypes has long been a fundamental element of the characterization of bacteria in diagnostic microbiology laboratories. Hierarchical typing based on genetic characters has only recently been applied, for classification of *Bacillus anthracis* and *Francisella tularensis* strains [1], [24], [34], [35]. This work demonstrates that a genetic hierarchical approach, based on carefully selected markers with canonical properties, can be used across an extensive phylogenetic typing range in the genus *Francisella*.

We have identified 34 genomic markers serving as phylogenetic guides, which can be added to or excluded from an assay depending on the testing objectives, i.e. according to the taxonomic and geographical resolution required. For example, in diagnostics, where the purpose is to verify the presence or absence of *F. tularensis* specimens, including canonical markers for species and subspecies levels in the assay may be sufficient. In contrast, in epidemiological investigations, where the aim is to track disease-transmission paths and/or sources, higher typing resolution might be desired, and thus markers that characterize the complete phylogeny, or alternatively only a selected subset with high resolution, should be included in the assay. In forensic investigations, complete characterization of isolates is needed to provide statistical and unambiguous evidence to infer relationships between isolates, and thus all canonical markers may be included in the assay. Geographical aspects could also be taken into consideration when selecting markers to be included. For example, in clinical laboratories located in Scandinavia it is not expected to find *F. tularensis* subsp. *tularensis* isolates in clinical samples tested, since this subspecies is confined to North America. Thus, only one canonical marker specific for the subspecies *tularensis* may be included and not all markers characterizing subclades of the subspecies. Instead, a very high discriminatory power for all the *F. tularensis* subsp. *holarctica* genetic groups that are known to be present in Sweden would be desired, i.e., groups B1 to B4 in Figure 1. Therefore, all canonical markers defining these subclades may be included. Finally, since we have included genomic markers for discriminating human pathogenic *F. tularensis* isolates from *F. philomiragia* and *F. novicida* which are of less clinical relevance and often present in environmental sources, the assay could potentially be used to monitor environmental *Francisella*.

A comparison of results obtained from SNP and INDEL markers shows good agreement. Both marker types apparently provide similar and stable phylogenetic information. Further, INDELs and SNPs are slowly mutating markers that provide very similar typing resolution. The lower typing resolution of INDELs in our assay was probably due to marker discovery bias: INDELs were easier to identify in the relatively few and genetically diverse available genome sequences than in the many available short sequence stretches from closely related isolates. In contrast, SNPs could be readily identified in both kinds of DNA sequences. We note that INDEL markers in the real-time PCR assay strengthen the SNP marker information at the main phylogenetic nodes (Figure 3). Deletion events should be evolutionarily unidirectional [2], while SNPs may revert. Thus, SNPs may (at least theoretically) display homoplastic patterns, while INDELs should not do so in a clonally structured bacterial population. We found that use of INDELs made the assay more robust and provided additional resolution at nodes where no corresponding SNP was identified.

The limit of detection of our assay was 100 pg of DNA, based on the lowest amount of DNA from which all 34 markers included in a single plate were amplified; a higher quantity than minimum amounts reported for other real-time PCR assays with fewer targets. This is a limitation that should be addressed in future work. Possibly, adaptation to a real-time PCR system including probes such as TaqMan SNP Genotyping Assay or the SNaPshot (Single Nucleotide Primer Extension) Assay (Applied Biosystems), could provide higher sensitivity. However, the reproducibility of the results was good and the failure of classification low, indicating that the assay was technically robust. The applicability of our assay to clinical isolates was also demonstrated, since we were able to characterize *F. tularensis* subsp. *holarctica* isolates obtained from patients in Sweden 2008, and bacterial DNA in ulcer specimens from tularemia patients.

We observed that the isolate FSC186 was classified as belonging to MLVA group B3 by Johansson et al 2004, but our data based on slowly mutating canonical SNPs and INDELs indicate that the isolate belongs to group B1. This finding illustrates the risk of homoplastic effects when using very rapidly mutating genetic markers in the MLVA for *F. tularensis* (Table 6). A detailed analysis showed that the MLVA markers Ft-M3 and Ft-M6 were the causes of the homoplasy effect. Accordingly, a genetic analysis of *F. tularensis* isolates including Ft-M3 and Ft-M6 should be complemented with analysis of more robust markers, such as SNPs and/or INDELs to ensure correct phylogenetic classification.

In summary, real-time PCR assays based on a hierarchical classification concept, as exemplified in this work, are flexible typing tools for phylogenetic and geographical resolution of *Francisella.* The level of discrimination can be easily adjusted by adding or removing genetic markers, a property which is not generally provided by conventional PCR methods or by previously developed real-time PCR assays. The presented hierarchical real-time PCR array could be used in public health laboratories as well as in research laboratories for a wide range of *Francisella* identification and typing purposes.
